# Reprogramming Antitumor Immunity: NK Cell Strategies to Navigate the Immunosuppressive Tumor Microenvironment

**DOI:** 10.1002/advs.75326

**Published:** 2026-04-21

**Authors:** Tereza Kochs, Pranay Nath, Alice Chen, Rizwan Romee, Andreia Maia

**Affiliations:** ^1^ Medical Oncology Department Dana‐Farber Cancer Institute Harvard Medical School Boston Massachusetts USA

**Keywords:** CAR‐NK cells, cell therapy, immunotherapy, in vivo gene editing, natural killer cells, tumor immune escape

## Abstract

Tumor immune escape is a major barrier to durable cancer immunotherapy, as advanced malignancies create a tumor microenvironment (TME) that preferentially exhausts and disables T cell responses. While most approved cell therapies are T cell‐based, this limitation motivates the exploration of an alternative effector cell platform. Natural killer (NK) cells, innate cytotoxic lymphocytes capable of antigen‐independent recognition and killing, offer a compelling foundation for next‐generation therapies with an improved safety profile. In this review, we first outline the cellular, molecular, and metabolic features of the immunosuppressive TME that restrict cytotoxic lymphocyte function, emphasizing mechanisms that limit immune cell‐mediated responses. We then summarize key aspects of NK cell biology that can circumvent these barriers and critically evaluate current NK‐based strategies, including engineered chimeric antigen receptor (CAR)‐NK products and metabolic and trafficking interventions. Finally, we highlight emerging in vivo viral and mRNA/lipid nanoparticle platforms for CAR‐NK generation and their potential to enhance scalability and therapeutic durability.

## 1 Introduction

As malignancies evolve, they adopt strategies to evade immune surveillance [1]. The “3 C's” model of immune evasion outlines the strategies employed by tumor cells: *camouflage* (evading immune detection), *coercion* (reprogramming native immune cells to support tumor growth), and *cytoprotection* (developing intrinsic resistance to immune‐mediated killing) [2]. While the specific tactics may vary by malignancy, they collectively compromise the efficacy of the immune response, and by extension, the immunotherapeutic approach [3]. Notably, T cells, a common platform for adoptive therapy, become exhausted rapidly within the tumor microenvironment (TME), demonstrating the need for exploration into alternative approaches [4]. Natural killer (NK) cells, as key effectors of the innate immune system, offer a compelling alternative, providing a critical first line of defense against malignant and virus‐infected cells, owing to their capacity for rapid target recognition and potent cytotoxicity [5, 6]. Although NK cells deploy cytotoxic mechanisms similar to those used by adaptive immune cells, including T lymphocytes, they lack antigen‐specific receptors and do not require priming through antigen presentation by antigen‐presenting cells (APCs) [6, 7], which makes NK cells an attractive platform for the development of effective immunotherapies. In this review, we first examine the cellular and molecular landscape of the immunosuppressive TME, detailing how infiltrated regulatory cells and inhibitory factors orchestrate cytotoxic immune cell dysfunction. We then provide a brief overview of NK cell biology and evaluate current platforms for NK cell therapy. Finally, we discuss the future of the field, highlighting next‐generation strategies such as the in vivo generation of chimeric antigen receptor (CAR)‐NK cells using mRNA‐lipid nanoparticles (LNPs) or viral‐based approaches designed to overcome tumor escape and enhance therapeutic efficacy.

## 2 The Immunosuppressive TME as a Major Barrier to Immunotherapy

Solid tumors establish a profoundly immunosuppressive TME that limits the activity, persistence, and trafficking of cytotoxic lymphocytes, including CD8^+^ and CD4^+^ T cells, and NK cells, thereby constraining the efficacy of immunotherapy [8, 9]. Cancer‐associated fibroblasts (CAFs) remodel the extracellular matrix (ECM) and secrete transforming growth factor‐β (TGF‐β) and other inhibitory cytokines, creating dense collagen‐ and fibronectin‐rich stroma and exclusion zones that correlate with poor prognosis and reduced lymphocyte infiltration [10, 11, 12, 13].

Myeloid‐derived suppressor cells (MDSCs) and tumor‐associated macrophages (TAMs) further reinforce immunosuppression by producing prostaglandin E2 (PGE2), IL‐10, and TGF‐β, depleting nutrients through arginase and Indoleamine‐2,3‐dioxygenase (IDO) activity, and generating reactive oxygen species (ROS) [14, 15], while hypoxia‐driven HIF‐1α/STAT3 signaling promotes their differentiation and pro‐angiogenic functions [16, 17]. TAM‐derived chemokines, such as CCL20, recruit regulatory T cells (Tregs), establishing a self‐reinforcing suppressive circuit within the TME [18]. In parallel, tumor‐derived IL‐10, IL‐35, TGF‐β, and vascular endothelial growth factor (VEGF) impair antigen presentation [19, 20, 21], reduce IL‐2 responsiveness [22], reduce activating receptor expression on NK and T cells [23, 24], and downregulate endothelial adhesion molecules such as vascular cell adhesion molecule‐1 (VCAM‐1) and intracellular adhesion molecule‐1 (ICAM‐1), thereby limiting effector cell activation and trafficking into tumors [25].

Metabolic suppression represents a convergent mechanism of immune dysfunction, as IDO‐mediated tryptophan depletion and kynurenine accumulation, extracellular adenosine signaling via A2A/A2B receptors, lactate‐induced acidosis, and arginine depletion collectively blunt interferon‐γ (IFN‐γ) and tumor necrosis factor (TNF) production [26, 27, 28, 29], disrupt mitochondrial function, thereby rendering NK and other immune cells dysfunctional [30, 31], and destabilize immune signaling, interfering with CD3ζ, ZAP‐70, and MAP kinase pathways in T and NK cells [32, 33]. In addition, both T and NK cells upregulate multiple immune checkpoint pathways, including PD‐1, CTLA‐4, LAG‐3, TIM‐3, and TIGIT, which enforce exhaustion programs [34, 35, 36] regulated by transcriptional factors such as TOX, TCF‐1, and NR4A family members [37, 38]. Although some studies report minimal or absent PD‐1 expression on NK cells, others demonstrate that increased PD‐1 expression correlates with NK cell dysfunction [39]. These pathways limit the durability and efficacy of checkpoint blockade and cell therapies, particularly in solid tumors [40].

In contrast, the liquid tumor microenvironment is dynamic and circulating, with unique modalities that present immunosuppressive hurdles. Circulating tumor cells (CTCs) are taxied alongside immune system components, such as lymphocytes, platelets, neutrophils, and other immune cells that provide shielding from immune recognition [41, 42]. Immunosuppressive signaling molecules such as TGF‐β and IL‐10 dampen the efficacy of any launched immune response [43]. Within immune organs, hematological malignancies are supported by a network of stromal, TAM, and tumor‐associated immune cells that establish interconnected signaling networks promoting immunosuppression [44]. Circulating tumor DNA (ctDNA), RNA, and vesicles compose a molecular “circulome,” which can often shuttle immunomodulatory cargo such as checkpoint ligands and immunosuppressive cytokines [45]. The bone marrow niche serves a key immunosuppressive role within liquid tumors, acting as a hub for immunosuppressive factors; bone marrow stromal and endothelial cells produce chemokines such as CXCL12 that not only mediate tumor cell homing but also recruit Tregs and exclude effector lymphocytes, while direct cell‐cell contact and soluble factors induce T cell exhaustion and NK cell dysfunction [46, 47].

Collectively, the TME imposes physical, metabolic, and inhibitory signaling barriers that converge on effector cell dysfunction [48]. These constraints provide a rationale for advancing current cellular therapies by leveraging multiple facets of immune cell biology and engineering effector cells to enhance their resilience to TME‐mediated suppression. These constraints provide a rationale for therapeutic strategies that deploy immune cells capable of antigen‐independent cytotoxicity and enhance resilience to TME‐mediated suppression [49]. In this context, NK cells emerge as a promising platform, especially when equipped with targeted engineering strategies to withstand immunosuppressive factors, metabolic stress, and inhibitory checkpoint signaling [50].

## 3 Use of NK Cells as an Effective Immune Therapy

**FIGURE 1:**
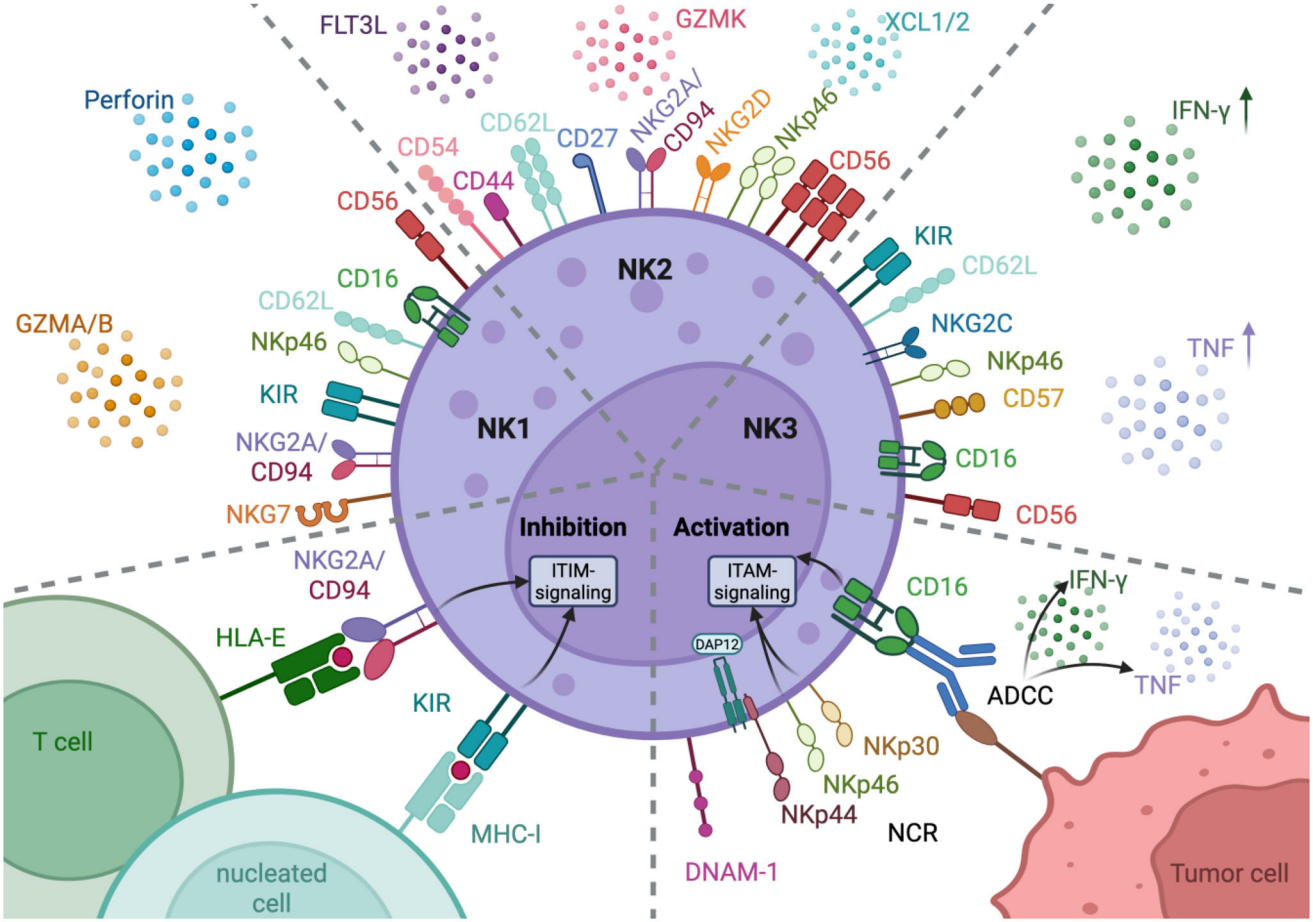
NK cell subsets and functions. Human NK cells can be broadly classified into cytotoxic NK1 (CD56^dim^ CD16^+^), regulatory NK2 (CD56^bright^ CD16^−^), and memory‐like NK3 (CD56^dim^ CD16^+^ CD57^+^ NKG2C^high^), as shown in the top part of the figure. Key NK cell activation pathways include ITAM‐dependent signaling downstream of CD16 mediating antibody‐dependent cell cytotoxicity (ADCC) and by engagement of natural cytotoxicity receptors (NCRs) like NKp30, NKp44, and NKp46 (lower right). Major inhibitory pathways include signaling driven by ITIM engagement following recognition of MHC‐I molecules by inhibitory KIRs and HLA‐E by NKG2A/CD94 complex (lower left). This figure was created with BioRender.

NK cells are innate lymphocytes that recognize and eliminate malignant and virus‐infected cells without prior priming [51, 52]. They arise from common lymphoid progenitors, but, unlike T cells, do not undergo T cell receptor (TCR) rearrangement or express CD3, instead relying on germline‐encoded receptors for antigen‐independent activation [6, 7]. Human NK cells are broadly categorized into three subsets based on CD16, CD56, and CD57 expression, and functional specialization: cytotoxic NK1 (CD56^dim^ CD16^+^), cytokine‐producing NK2 (CD56^bright^ CD16^−^), and adaptive‐like NK3 (CD56^dim^ CD16^+^ CD57^+^ NKG2C^high^) (Figure 1) [53, 54].

NK1 cells predominate in the peripheral blood (PB) (∼ 90%), bone marrow, and spleen [55], and are optimized for direct tumor killing through high basal levels of perforin, granzymes, and NK cell granule protein 7 (NKG7) [54]. NK2 cells are enriched in lymphoid and mucosal tissues [5, 55], express activation and homing molecules such as CD27, NKG2D, NKp46, CD44, and CD62L [54], and secrete FLT3L and XCL1/2 to support dendritic cell (DC) recruitment and cross‐priming [54, 56, 57, 58, 59, 60]. In addition, NK2 cells express granzyme K (GZMK), enabling caspase‐independent cytotoxicity via complement activation [61]. NK3 cells acquire memory‐like properties after viral or bacterial stimulation, undergoing CD57‐associated epigenetic remodeling that confers enhanced recall responses and augmented antibody‐dependent cytotoxicity (ADCC), features now being explored for durable anti‐tumor immunity [54, 62].

NK effector function is regulated by the balance of activating and inhibitory signals [63]. NK cells preferentially target “missing‐self” cells with reduced MHC‐I expression [64], where relief of inhibitory killer‐cell immunoglobulin‐like receptor (KIR) and NKG2A signaling permits activation via NKG2D, NKp46, DNAM‐1 [65]. KIRs and CD94/NKG2A recognize classical and non‐classical MHC‐I molecules and transduce ITIM‐mediated inhibitory signals via SHP‐1/2 to maintain self‐tolerance, with subset‐specific expression patterns fine‐tuning cytotoxic thresholds across NK1, NK2, and NK3 subsets [66, 67].

Among activating receptors, CD16 (FcγIIIA) is central to mediating ADCC, coupling therapeutic or endogenous IgG [68, 69, 70, 71] to ITAM‐based CD3ζ/FCɛRIγ signaling and driving degranulation and pro‐inflammatory cytokine release [72, 73]. Natural cytotoxicity receptors (NCRs), including NKp30, NKp44, and NKp46, provide MHC‐independent tumor recognition [6, 74]; NKp30 or NKp46 signal via CD3ζ/DAP12 [40, 75, 76], and can also mediate crosstalk with APCs through B7‐H6 [6, 74, 77, 78], whereas NKp44 is restricted to activated NK cells and signals through DAP12 homodimers [6, 79, 80].

Cytokine exposure can imprint durable functional states, and cytokine‐induced memory‐like (CIML) NK cells generated by brief IL‐12/IL‐15/IL‐18 stimulation, followed by rest, exhibit stable chromatin remodeling [81, 82], enhanced metabolic fitness, and superior IFN‐γ production, and ADCC against leukemia and solid tumors in preclinical and early clinical studies [81, 83].

### 3.1 NK Cell‐Based Cellular Therapy and Sources

NK cells possess key therapeutic advantages over T cells, including rapid MHC‐independent cytotoxicity mediated by NCRs, NKG2D, and missing‐self recognition [5, 84], coupled with a lower propensity for causing graft‐versus‐host disease (GVHD) and a more favorable cytokine release syndrome (CRS) and neurotoxicity safety profile [85, 86]. Nonetheless, tumors can escape NK surveillance by shedding activating ligands such as MHC‐I chain‐related proteins A and B (MICA/B) or by upregulating inhibitory signals [87], processes driven in part by IFN‐γ‐dependent MHC‐I induction and TGB‐β‐mediated NKG2D downregulation [84, 88]. These evasion mechanisms underscore the need for next‐generation NK cell engineering strategies, including CAR‐NK therapy and in vivo modulation approaches, to enhance persistence, trafficking, and cytotoxicity, particularly within immunosuppressive solid TMEs.

Multiple cellular platforms have been developed for NK cell‐based therapy, each with distinct trade‐offs in potency, scalability, and clinical feasibility [89]. Peripheral blood (PB) NK cells provide mature NK1 and NK3 subsets with clinical safety data [6, 86] but limited proliferative capacity and relatively more difficult to genetically engineer compared to T cells [90]. Umbilical cord blood (CB) offers CD34^+^ progenitors with higher proliferative potential, used directly or via CB‐derived hematopoietic stem and progenitor cells (HSPCs), but requires prolonged and complex ex vivo expansion and differentiation protocols [91, 92, 93, 94]. Induced pluripotent stem cells (iPSCs) enable standardized and scalable NK cell products and facile genetic engineering [95], yet often yield an immature NK phenotype that demands maturation and activation protocols [89]. Finally, the NK‐92 cells are easily engineered and expanded “NK‐like” lymphoma cell line, but require irradiation prior to infusion due to their tumor origin, limiting their in vivo persistence [94, 96].

For therapeutic dosing, NK cell‐based therapies typically require 5 × 10^6^ to 1 × 10^8^ NK cells per kg [97], requiring robust ex vivo expansion platforms. K562‐derived feeder cells expressing membrane‐bound (mb) IL‐15 and 4‐1BBL can markedly outperform soluble cytokines in expanding CD3^−^ CD56^+^ NK cells, but are associated with telomere shortening and reduced persistence [98, 99]. Incorporation of mbIL‐21 activates STAT3, which then activates human telomerase reverse transcriptase (hTERT), allowing massive expansion [100, 101], improved activation marker expression, and mitigation of telomere erosion compared to mbIL‐15 alone [102, 103]. Combining K562‐4‐1BBL‐mbIL‐21/‐15 feeder systems supports rapid, large‐scale NK production and shapes metabolic and proliferative fitness, providing a practical backbone for current next‐generation engineered NK and CAR‐NK products [104].

## 4 Strategies to Enhance NK Cell‐Based Immunotherapy

### 4.1 CAR‐NK Cell Engineering

NK cells engineered to express CAR combine synthetic, antigen‐specific recognition with innate tumor surveillance mediated by germline‐encoded receptors such as NKG2D, DNAM‐1, and NCRs [85]. This dual targeting enables CAR‐NK cells to eliminate tumor cells via CAR‐dependent and CAR‐independent mechanisms, potentially reducing the risk of antigen loss‐driven escape that frequently hampers CAR‐T cell therapies [105]. Clinically, CAR‐NK cells have shown a favorable safety profile, with low incidence of CRS and neurotoxicity, lack of GVHD, and suitability for off‐the‐shelf allogeneic use without stringent HLA matching [4, 86, 106].

While CAR architecture was originally optimized for T cells, effective CAR‐NK design requires adapting these principles to NK‐specific signaling biology. A typical CAR comprises a single‐chain variable fragment (scFv) antigen‐binding domain linked via a spacer and transmembrane region to intracellular signaling modules [107, 108]. First‐generation CARs used CD3ζ alone and proved insufficient in vivo due to a lack of costimulation and poor expansion [107, 108], leading to second‐generation designs that coupled CD3ζ to a single costimulatory domain such as CD28 or 4‐1BB, which substantially improved proliferation and persistence in T cells [108]. Third‐generation CARs incorporate two costimulatory domains (e.g., CD28/4‐1BB or OX40/ICOS) to provide complementary signals [108]. For NK cell applications, both T cell‐derived costimulatory motifs (CD28, 4‐1BB) and NK‐specific signaling components are being explored (Table 1). CAR‐NK constructs include NK‐tailored modules such as 2B4, DAP12, and DAP10 alone or in combination with CD28 or 4‐1BB, can better harness endogenous NK cell activation pathways and have shown enhanced cytokine production, degranulation, and persistence in preclinical models [109, 110]. Modular designs that couple CAR signaling to IL‐15 or to NK‐specific adaptor signaling (e.g., NKG2D‐DAP12 fusions) are particularly promising for sustaining function in TME‐like conditions [111, 112].

**TABLE 1:** Summary of the key CAR‐NK signaling domains.

Target antigen	Costimulatory module(s)	Cancer type	References
CD19	CD28	B‐cell acute lymphoblastic leukemia (B‐ALL)	Liu, Enli et al. [113]
CD19	4‐1BB	Non‐Hodgkin lymphoma	Lei, Wen et al. [217]
CD123	CD28	Acute myeloid leukemia (AML)	Zhang, Leisheng et al. [218]
CD33	4‐1BB	AML	Huang, Ruihao et al. [219]
HER2	CD28	Glioblastoma	Strassheimer, F et al. [220]
NKG2D ligands	DAP10	Multiple solid tumors	Tang, Shuqing et al. [221],Li, Bin et al. [222]
NKG2D	2B4	AML	Li, Ye et al. [223]

Clinically, a landmark phase I/II study using CB‐derived CD19 CAR‐NK cells (incorporating IL‐15 and inducible caspase‐9) in relapsed non‐Hodgkin lymphoma and chronic lymphocytic leukemia (CLL) reported a 73% overall response rate with no severe CRS, neurotoxicity, or GVHD, underscoring the therapeutic potential and safety of this platform [86, 113]. More recent trials and preclinical studies are extending CAR‐NK therapy to solid tumors (Table 2), targeting antigens such as HER2, EGFR, GD2, EpCAM, and MUC1 in breast, ovarian, colorectal, neuroblastoma, and glioblastoma (GBM) models [114]. However, antigen heterogeneity, poor tumor infiltration, and TME suppression continue to limit efficacy, highlighting the need to integrate CAR design with TME‐resistance, metabolic armoring, and trafficking strategies to further improve their efficacy. Due to the highly heterogeneous antigenic landscape of solid tumors, single‐target CAR‐NK constructs can drive immunoediting by selectively eliminating antigen‐high cells but allowing the expansion of antigen‐low variants that evade CAR NK recognition [84]. Furthermore, even when CAR NK cells manage to infiltrate the dense ECM and irregular vasculature characteristic of many solid tumors, the immunosuppressive TME rapidly impairs their function with factors such as TGF‐β and adenosine, rendering the therapy ineffective over time and hence more difficult to achieve a robust clinical response [8].

**TABLE 2:** Overview of Clinical Major Trials Evaluating CAR‐NK Cell Therapy.

Disease	Target antigen	Phase	NK cell drug candidate	NCT no.
AML	NKG2D	I	Anti‐NKG2DL CAR‐NK	NCT05247957
AML	NKG2D	I	Anti‐NKG2DL CAR‐NK	NCT04623944
AML	CD33	I	anti‐CD33 CAR NK cells	NCT05008575
AML	CD33	I	Anti‐CD33/CLL1 CAR‐NK	NCT05215015
AML	CD33	I	anti‐CD33 CAR NK cells	NCT05601466
AML	CD70	II	CAR.70/IL15‐ NK cells	NCT05092451
ALL	CD19	I	Anti‐CD19 CAR‐NK cells	NCT05563545
B‐ALL	CD19	I	Anti‐CD19 CAR‐NK cells	NCT00995137
B‐ALL	CD19	I	Anti‐CD19 CAR‐NK cells	NCT05379647
B‐ALL	CD19	I	Anti‐CD19 CAR‐NK cells	NCT04796675
B‐ALL	CD19	II	iC9/CAR.19/IL15‐NK Cells	NCT03056339
B‐ALL	CD19	II	Anti‐CD19 CAR‐ITNK cells	NCT04747093
B‐ALL	CD19	II	Anti‐CD19 CAR‐NK cells	NCT05570188
BCL	CD19		Anti‐CD19+IL‐15 CAR‐NK cells	NCT03774654
BCL	CD19	I	Anti‐CD19 hnCD16+IL15 CAR NK (FT596)	NCT04245722
MM	BCMA	I	Anti‐BCMA CAR‐NK (FT576)	NCT05182073
H&N	PD‐L1	II	PD‐L1 CAR‐NK	NCT04847466
Ovarian	CLDN6	II	CLDN6‐CAR‐NK	NCT05410717
Lung cancer	MSLN, EGFR, HER2/ERBB2	I/II	Dual‐target CAR‐NK cells	NCT07467863
Breast cancer	HER2/ERBB2, MUC1, ROR1	I/II	Dual‐target CAR‐NK cells	NCT07486089
Colorectal cancer	CEA, GUCY2C, HER2	I/II	Dual‐target CAR‐NK cells	NCT07462650
Urothelial carcinoma	Nectin‐4, HER2	I	Dual‐target CAR‐NK cells	NCT07492628
AML	CD123	I	anti‐CD123 CAR NK cells	NCT05574608
AML	CD123	I/II	anti‐CD123 CAR NK cells	NCT06006403
AML	CD123	I	anti‐CD123 CAR NK cells	NCT06690827
AML	CD123	I	anti‐CD123 CAR NK cells	NCT06201247

ALL = acute lymphoblastic leukemia;

AML = acute myeloid leukemia;

B‐ALL = B‐cell acute lymphoblastic leukemia;

BCL = B cell lymphoma;

MM = Multiple myeloma;

H&N = Head and neck.

### 4.2 Novel Immune Masking Strategies as Critical Enablers of Universal, Off‐the‐Shelf Allogeneic NK Cell Therapies

Universal off‐the‐shelf allogeneic NK cell products are particularly attractive for immunotherapy because they mitigate potential delays of patient‐specific manufacturing, ensure consistent product quality across batches, and substantially reduce cost and logistical complexity, all key barriers that currently limit access to cell therapy [106, 115, 116]. Like any other cell product, allogeneic NK cells are susceptible to the host‐versus‐graft rejection, which remains a major barrier to their durable persistence after adoptive transfer [117, 118]. Recent advances in genome engineering now allow selective disruption of HLA A, B, and C molecules and expression of PDL‐1 to reduce host T cell recognition, while simultaneously expressing non‐classical HLA molecules such as HLA‐E to inhibit host NK cells [119]. Similar CRISPR/Cas9‐mediated knockout of adhesion ligands CD54 and CD58, along with B2M in iPSC‐derived CAR‐NK cells, protected them from host NK cell and T cell‐mediated rejection without compromising antitumor activity, thus supporting a potential strategy for universal immune‐evasive cell therapies [120].

### 4.3 Overcoming TME Suppression and Metabolic Dysfunction

**FIGURE 2:**
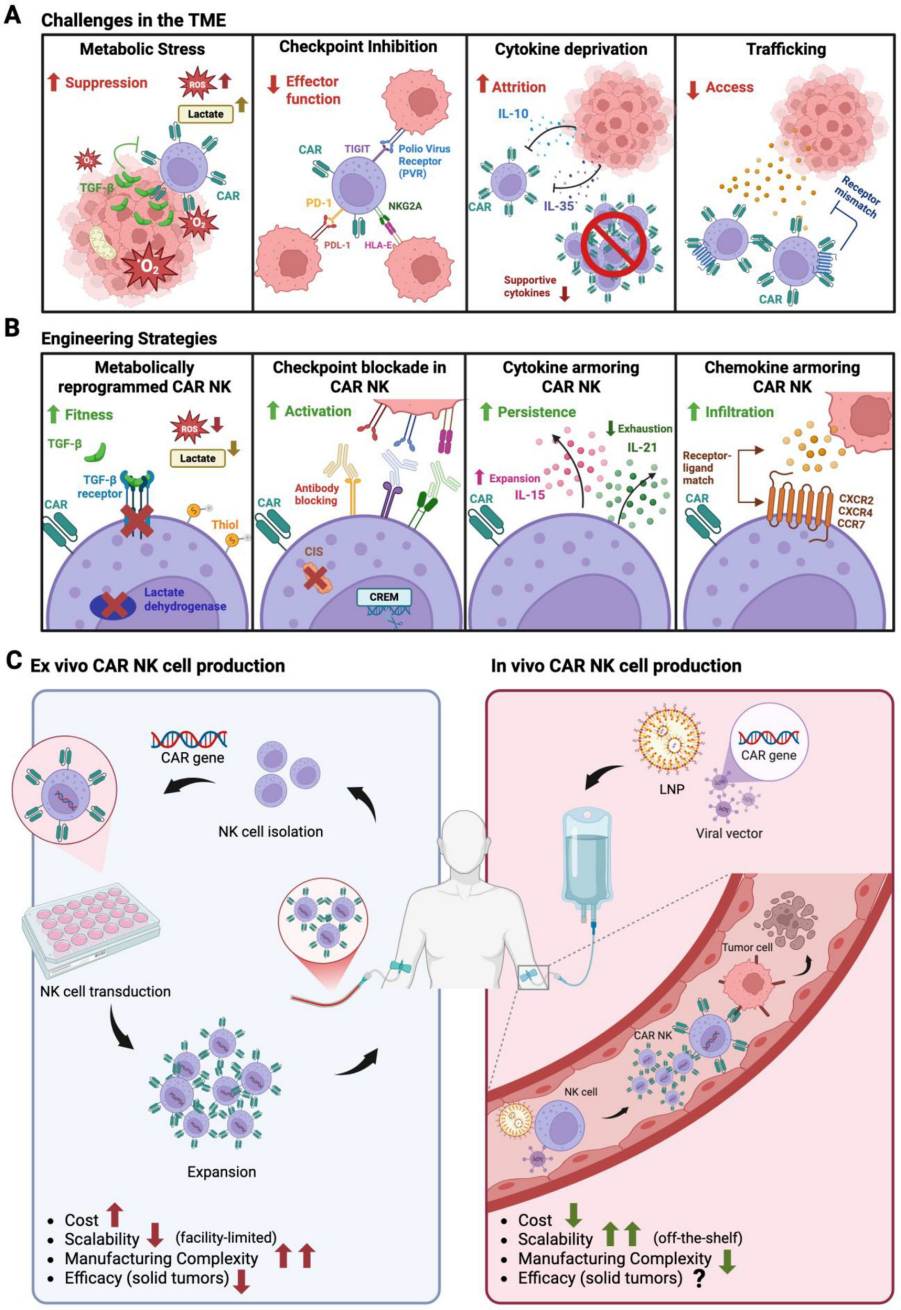
Engineering strategies to enhance NK cell function within the TME. (A) Key limitations of current chimeric antigen receptor (CAR)‐NK cell‐based therapies imposed by the TME, (B) together with representative engineering strategies designed to alleviate metabolic stress, overcome inhibitory checkpoint signaling, prevent cytokine deprivation, and enhance tumor trafficking. (C) Comparative overview of ex vivo versus in vivo engineered CAR NK cell approaches, highlighting differences in cost, scalability, manufacturing complexity, and potential therapeutic efficacy. This figure was created with BioRender.

TGF‐β is abundantly secreted by tumor cells, CAFs, and myeloid cells in solid tumors, including hepatocellular carcinoma (HCC), pancreatic ductal adenocarcinoma (PDAC), GBM, and breast cancer, suppressing NK cell proliferation, NKG2D expression, and cytotoxicity [121, 122, 123, 124] (Figure 2A,B). To counter this, several engineering strategies have been developed, including expression of dominant‐negative TGF‐β receptors (dnTGFβRII) that lack the intracellular kinase domain and block SMAD signaling [125, 126, 127, 128], CRISPR‐mediated TGFBR2 knockout in iPSC‐derived NK cells to improve NK anti‐tumor function [121, 129], and TGF‐β switch receptors that fuse the extracellular TGF‐β‐binding domain to activating modules such as DAP12 [130], thereby converting an inhibitory cue into a stimulatory signal that enhances NK cell cytotoxicity in high TGF‐β environments.

Additionally, the hypoxic and metabolically dysregulated TME generates additional suppressive factors, including lactate and ROS [131, 132] (Figure 2A,B). Lactate accumulation reduces IFN‐γ production and impairs NK degranulation [133]; to overcome this, monocarboxylate transporter (MCT4) has been inhibited to improve NK cell function [134] and lactate dehydrogenase A (LDHA) has been genetically ablated in NK cells, enhancing lactate oxidation and limiting intracellular acidosis [133, 135] ROS produced by tumor cells and MDSCs induce oxidative damage and metabolic dysfunction in NK cells [136, 137]. IL‐15 priming activates an mTOR‐driven thioredoxin‐1 (Trx1) antioxidant program that preserves thiol density on the NK cell surface and protects both NK and neighboring T cells from ROS‐mediated apoptosis [138]. In parallel, strategies targeting adenosine (A2A receptors or CD39/CD73) and IDO‐mediated kynurenine production, for example, which were initially developed in T cell and CAR‐T settings [139, 140, 141, 142, 143], are being adapted to NK cells to relieve metabolic checkpoint pressure.

Checkpoint and inhibitory receptor pathways also contribute to NK dysfunction in hematological and solid tumors [144] (Figure 2A,B). Genetic disruption or blockade of PD‐1, TIGIT, NKG2A, and FAS on NK cells has been shown to enhance degranulation, cytokine production, viability, and persistence in preclinical models [145, 146, 147, 148], and checkpoint‐edited CAR‐NK cells incorporating NKG2A, CISH, or CREM KO show promising preclinical efficacy [149, 150, 151, 152]. Genome‐wide CRISPR screens performed under TME‐mimetic conditions have also identified intrinsic NK checkpoints, such as Mediator Complex Subunit 12 (MED12), Ariadne RBR E3 Ubiquitin Protein Ligase 2 (ARIH2), and Cyclin C (CCNC), whose genetic KO enhances metabolic fitness and improves in vivo tumor control [153, 154], suggesting genetically optimized backbones for next‐generation CAR‐NK products. Complementing these loss‐of‐function approaches, Yang et al. Identified Olfactory receptor family 7 subfamily 10 (OR7A10) as a gain‐of‐function target, demonstrating that its co‐expression with HER2‐CAR potentiates anti‐tumor activity, elevated IFN‐γ and TNF production, mitigates exhaustion, and promotes a favorable survival profile characterized by increased GZMB expression, upregulation of B‐cell Lymphoma‐2 (BCL2) and BCL2‐like 1 (BCL2L1), and reduced BAX expression [155].

Targeted immunodepletion of suppressive stromal populations, particularly CAFs, can disrupt immunosuppressive signaling networks and physical barriers within the tumor microenvironment, thereby enhancing cytotoxic activity of T cells and NK cells via increased rates of infiltration [156]. This strategy is further supported by broader evidence that removing or reprogramming suppressive components of the TME restores antitumor immunity and improves the efficacy of cell‐based immunotherapies [157]. Together, these receptors, metabolic, cellular, and checkpoint engineering approaches illustrate a modular toolkit to preserve or even enhance NK cell function within immunosuppressive TME and provide a foundation for combining CAR‐NK therapy and microenvironment‐targeted interventions.

### 4.4 Cytokine and Chemokine Armoring

CAR‐NK cells must maintain metabolic fitness and adequate survival in nutrient‐deprived and hypoxic TME, where glucose and amino acid scarcity impair ATP‐dependent degranulation and promote premature senescence [158] (Figure 2A,B). Cytokine armoring, whereby CAR‐NK cells are engineered to express cytokines, such as IL‐15 or IL‐21, provides autocrine and paracrine support that sustains proliferation, survival, and metabolic reprogramming toward enhanced glycolysis and oxidative phosphorylation, reducing dependence on exogenous cytokines [159, 160, 161, 162, 163]. IL‐15 has emerged as the most powerful armoring, as it activates the JAK‐STAT pathway that enhances NK persistence without the Tregs expansion associated with IL‐2 [164, 165]. IL‐15‐armored CAR‐NK cells have shown improved persistence and anti‐tumor activity in hematologic models and are now being evaluated in clinical trials [166, 167, 168]. In settings where cytokine arming is limited by systemic toxicity, alternative strategies to exploit IL‐15R signaling have been explored [169]. Deletion of Aih2 and Ube2f enhances IL‐15Rß expression on NK cells, thereby promoting anti‐tumor immunity through IL‐15‐mediated activation of JAK1/3‐STAT5 signaling and improving survival, proliferation, and effector function without increasing cytokine levels but rather elevating receptor density [170]. On the other hand, IL‐21 armoring enhances anti‐GBM efficacy and induces memory‐like properties of NK cells, promoting resistance to exhaustion [171, 172, 173].

Chemokine armoring further improves NK cell infiltration and trafficking into tumor sites by matching NK chemokine receptor expression to tumor‐derived chemokines (Figure 2A,B). Engineered NK cells with CXCR2 enhance homing to CXCL1/8‐rich renal cell carcinoma (RCC) [174], whereas CXCR4 expression promotes migration into CXCL12‐rich glioblastoma cells and myeloma bone marrow [175, 176] with combinatorial CXCR4/CCR7 further enhancing migration [177]. Armoring strategies combining cytokine secretion with chemokine receptor modification have also been explored, such as engineering NK cells to express IL‐15 with CCL21 or IL‐2 with CXCR2 to achieve stronger cytotoxicity [178, 179]. Collectively, these metabolic, cytokine, trafficking, and genetic engineering strategies are beginning to overcome key TME barriers and will underpin future platforms that extend in vivo NK cell engineering approaches to expand the reach and durability of NK‐based immunotherapy.

## 5 Future Directions: In Vivo CAR‐NK Engineering

Current ex vivo CAR‐NK manufacturing relies on specialized Good Manufacturing Practice (GMP) facilities, complex logistics, extended production timelines, and costs estimated at several hundred thousand dollars per patient, thereby constraining global accessibility [180]. In vivo CAR engineering represents a fundamentally different paradigm, in which CAR constructs are delivered directly to immune cells within the patient using viral vectors or non‐viral platforms such as mRNA delivered using lipid nanoparticles (LNPs), enabling in situ generation of engineered effectors and the potential for repeat dosing [181] (Figure 2C).

In vivo viral delivery uses modified viruses to transduce circulating or tissue‐resident lymphocytes and integrate CAR transgenes into their genomes [182]. CD7‐targeted lentiviral vectors developed by Interius Biotherapeutics have demonstrated selective in vivo transduction of CD7^+^ T and NK cells, enabling CAR expression without apheresis [183]. Umoja Biopharma's VivoVec lentiviral platform combines cocal fusion glycoproteins with CD80/CD58 to selectively expand CAR‐T cells in vivo after systemic administration [184]. Additional programs, such as ESO‐T01, a lentiviral vector that uses a nanobody‐based targeting system, have reported encouraging early clinical data in relapsed/refractory multiple myeloma, with deep responses after a single infusion [185, 186, 187]. However, recent early clinical data suggest that in vivo CAR‐T approaches may be associated with substantial on‐target toxicities, underscoring the need for careful safety optimization and consideration of NK cells as an alternative effector cell type [188].

In addition, while viral vectors provide durable CAR expression, they face challenges from pre‐existing neutralizing antibodies, vector immunogenicity, and rapid clearance in immunocompetent hosts, emphasizing the need for rigorous evaluation in non‐human primate models with human‐like immunity. Pre‐existing neutralizing antibodies (NAbs) against adeno‐associated virus (AAV) capsid proteins could partially or completely inhibit transduction of target cells and narrow the pool of patients who have AAV antibodies in their bloodstream from participating in clinical trials concerning systemic delivery of AAV‐based therapies [189]. Beyond humoral immunity, viral vectors also trigger innate immune sensing, where AAV and lentiviral components are recognized and activate NK cells in an uncontrolled manner, accelerating the adaptive immune responses against capsid and transgene‐derived antigens, which shortens the therapeutic window [190]. Even so, vectors that clear these two barriers still face rapid phagocytic clearance, where roughly 20% of splenic macrophages and monocytes took up CD3‐targeted vectors, and low levels of CAR expression were observed on macrophages upon administration of lentiviral particles with anti‐CD3 antibodies [191]. This was also observed in one of the most advanced NK‐targeting lentiviral platforms to date, INT2104, where liver macrophage uptake was detected despite the vector's CD7‐targeting specificity [183]. Several engineering strategies have been proposed to mitigate these immune barriers, including capsid engineering for improved antibody evasion, transient immunosuppressive preconditioning with prednisolone and rapamycin to delay anti‐capsid IgG development, depletion of CpG to dampen innate sensing, and incorporation of CD47, a “don't eat me” signal onto vector surfaces to reduce phagocytosis [191, 192, 193, 194, 195]. However, these mitigation strategies remain mostly preclinical and have not been tested specifically in in vivo NK cell‐targeted delivery.

On the other hand, non‐viral platforms, particularly LNPs encapsulating CAR mRNA, have rapidly advanced as flexible tools for in vivo gene delivery [196, 197]. In a murine leukemia model, LNP encoding CD19‐CAR mRNA has generated functional CAR‐T cells in vivo and matched the therapeutic performance of adoptive T cell therapy [198, 199, 200]. Off‐the‐shelf anti‐CD19 CAR NK cells via macropinocytosis‐dependent mRNA delivery have also been developed and demonstrated superior transfection efficacy in NK cells over T cells and potent cytotoxicity against Raji cell lines and primary B‐ALL patient samples [201]. Additionally, BCMA‐directed LNPs have induced meaningful responses in relapsed or refractory MM, demonstrating feasibility in humans [202]. In a recent study, optimized mRNA‐LNP formulations achieved high transfection efficiency in NK‐92 cells, with the resulting BCMA CAR NK‐92 cells exhibiting >10% BCMA‐specific killing against myeloma lines, with ongoing work extending the platform to primary NK cells and in vivo models [202]. In addition, Dual‐payload LNPs co‐encapsulating a tumor‐specific CAR and IL‐7 mRNA have also further improved T cell expansion and anti‐tumor activity in melanoma models compared to CAR‐only constructs [203, 204, 205], illustrating how cytokine armoring can be implemented in vivo and potentially extended to CAR NK platforms as well. However, although preclinical work has shown that LNP‐mRNA can generate CAR‐NK cells in vivo [206], clinical translation of in vivo CAR‐NK engineering has not yet been achieved.

Several technical hurdles must be overcome to fully perform in vivo CAR‐NK therapy. Linear mRNA has a short intracellular half‐life (∼ 7 h), prompting the development of more durable RNA formats: circular RNA and self‐amplifying RNA can prolong CAR expression and reduce dosing requirements, and early data from platforms such as Orna Therapeutics indicate improved transfection efficiency and sustained protein production in vivo [206, 207, 208, 209, 210, 211]. NK‐specific targeting remains a central obstacle, as commonly used markers such as NKp46 and CD56 are shared with ILC subsets and other lineages [212, 213], complicating selective delivery. In parallel, the transient expression window of mRNA (4–5 days) [214] that suffices for T cell expansion and memory formation may be limiting for short‐lived NK cells (∼2 weeks), necessitating repeated dosing, self‐replicating RNA platform, or hybrid strategies that combine viral and non‐viral vectors [215]. Another crucial bottleneck is the inefficiency of endosomal escape to achieve sufficient cytosolic mRNA delivery, which is poorly characterized in primary NK cells as they primarily rely on macropinocytosis rather than receptor‐mediated endocytosis, which further complicates the design challenge [216].

In vivo CAR‐NK cell engineering has the potential to merge NK cells’ inherent safety profile and antitumor activity (low CRS and GVDH risk, MHC‐independent killing, and compatibility with universal donor platforms) with streamlined bedside manufacturing that circumvents the high cost and logistical burdens of ex vivo production. Progress in identifying NK‐specific targeting ligands, improving durability with advanced RNA platforms or vector designs, and minimizing immunogenicity will be pivotal for transitioning from labor‐intensive ex vivo products to globally scalable, in vivo‐based NK precision immunotherapies.

## 6 Conclusion

These insights highlight both the progress made and the persistent challenges in overcoming the immunosuppressive TME, characterized by a dense ECM, immunosuppressive cell populations, inhibitory cytokines and chemokines, and metabolic constraints. In this context, NK cells have emerged as a promising alternative to T cells for engineering immunotherapies, owing to their intrinsic cytotoxicity and suitability for off‐the‐shelf allogeneic applications. CAR‐NK cells, developed based on CAR‐T principles but adapted to NK cell biology, have shown heterogeneous outcomes in solid tumors, largely due to limited tumor infiltration and TME‐mediated suppression. To overcome these barriers, diverse engineering strategies have been explored, including rewiring inhibitory signaling, alleviating metabolic constraints, and targeting checkpoint receptors, alongside cytokine and chemokine armoring to enhance activation, trafficking, proliferation, and persistence. Despite these advances, ex vivo manufacturing remains complex and costly, driving interest in in vivo CAR delivery. Viral platforms enable durable expression through genomic integration, with efforts to evade pre‐existing immunity, whereas non‐viral approaches, such as LNP‐mediated delivery, are limited by transient expression. Ultimately, integrating precise genetic engineering with improved delivery strategies and a deeper understanding of NK cell biology will be essential to unlock the full therapeutic potential of CAR‐NK cells in solid tumors.

## Author Contributions

TK, PN, and AC contributed to conceptualization and writing; RR and AM contributed to conceptualization, supervision, and editing; all authors approved the final version of the manuscript.

## Conflicts of Interest

RR is cofounder of InnDura Therapeutics and serves on the scientific advisory board of Glycostem Therapeutics. None of the cited studies involves products from these companies. All other authors declare no competing interests.

## Data Availability

Data sharing not applicable to this article as no datasets were generated or analysed during the current study.
